# Mapping a Type-specific Epitope by Monoclonal Antibody against VP3 Protein of Duck Hepatitis A Type 1 Virus

**DOI:** 10.1038/s41598-017-10909-7

**Published:** 2017-09-07

**Authors:** Xiaoying Wu, Tingting Zhang, Fanyi Meng, Dongchun Guo, Xiuchen Yin, Shaozhou Wulin, Chenxi Li, Qingshan Zhang, Ming Liu, Yun Zhang

**Affiliations:** State Key Laboratory of Veterinary Biotechnology, Harbin Veterinary Research Institute of Chinese Academy of Agricultural Sciences, Harbin, 150001 China

## Abstract

Duck hepatitis A subtype 1 virus (DHAV-1) infection causes high mortality in ducklings, resulting in significant losses to duck industries. VP3 is a structural protein of DHAV-1. However, B-cell epitopes on VP3 have not been investigated. To stimulate VP3 antibody response, eukaryotic expression plasmid pCI-neo-VP3 was constructed and used as DNA immunogen to prepare mAbs. Western blot showed that 25.5 kDa VP3 could be detected by mAbs in duck embryo fibroblast (DEF) cells transfected with pCI-neo-VP3. Immunofluorescence assay showed that mAbs could specifically bind to DEF cells infected with DHAV-1. DAPI staining indicated that VP3 localizes to the cytoplasm and nucleus of DHAV-1 infected DEF. With neutralizing mAb 3B7, minimal epitope PSNI was mapped. Sequence alignment indicated that ^205^PSNI^208^ is highly conserved among DHAV-1, but different from those of DHAV-2 and DHAV-3. Epitope peptide reacted specifically with DHAV-1-positive duck sera by dot blotting, revealing PSNI is DHAV-1 type-specific epitope and the importance of these amino acids in antibody-epitope binding reactivity. These findings provided useful information for understanding the antigenicity of VP3 and might be valuable in the development of epitope-based vaccine or diagnostic kit for DHAV-1 infection and provide insights for understanding the pathogenesis of DHAV-1.

## Introduction

Duck hepatitis A virus (DHAV), known as original duck hepatitis virus type 1 (DHV-1), is a member of the genus *Parechovirus* in the family *Picornaviridae*, consisting of a single-stranded, positive-sense RNA. It is genetically divided into three serotypes: the original worldwide type 1 virus (DHAV-1)^1, 2^, serotype 2 isolated only in Taiwan (DHAV-2)^3^, and serotype 3 occurred in South Korea and China (DHAV-3)^4–6^. Duck hepatitis A type 1 virus causes a fatal and rapid spread of disease in ducklings primarily characterized by hepatitis^7^. DHAV-1 disease has spread worldwide and continues to be a threat to ducklings because of its high mortality.

The complete genome of the DHAV-1 strain is about 7.7 kb long, which is organized into a single, large open reading frame (ORF) flanked by 5′and 3′ untranslated regions. The ORF can be translated into 12 mature proteins, including structural (VP0, VP3, and VP1) and non-structural proteins (2A1, 2A2, 2A3, 2B, 2C, 3A, 3B, 3C, and 3D)^1, 3, 4^. The VP1 is the most external and most dominant of the picornaviruses surface protein, with the highest genetic diversity among isolates. Structural and antigenic studies indicated that VP1 of picornavirus contains critical antigenic determinants that are responsible for the induction of neutralizing antibodies^8, 9^. The VP1 of DHAV-1 has been proved to be the most reliable indicator for DHAV-1 infection^10^. Recently, it has been proved that VP1 of DHAV-1 involves receptor-binding activity and elicits neutralizing antibodies^11, 12^. Structural protein VP3 plays an important role in virulence and the 56th amino acid in VP3 is the critical determinant of foot-and-mouth disease virus (FMDV) virus plaque phenotype and pathogenicity^13, 14^.

Although the genomic organization of DHAV-1 is well defined, mAbs against VP3 protein have not been developed and their binding sites have not been investigated. In this study, we first developed mAbs against VP3 protein and then identified real epitope on VP3 with obtained mAb. The principal aim of this study was to obtain specific immune responses to epitope of VP3 protein of DHAV-1 generated *in vivo*. Eukaryotic expression plasmids not only express protein faithfully, but also induce strong specific immune responses in animals. Thus, a eukaryotic expression plasmid (pCI-neo-VP3) was constructed and used as DNA immunogen to prepare mAbs in mice and then the epitope of VP3 was identified. Sequence analysis and immunological assay confirmed that this epitope is DHAV-1 type specific and would be useful in development epitope-based diagnostic kit for DHAV-1 infections.

## Results

### pCI-neo-VP3 construction and VP3 sequence analysis

The 712 bp VP3-encoding gene was amplified and cloned into pCI-neo vector. To confirm the recombinant plasmids were correct, pCI-neo-VP3 were digested by *Xho* I and *Sma* I restriction analysis and then for nucleotide sequencing. Electrophoresis results revealed that pCI-neo-VP3 plasmids were digested into two fragments, which were consistent with sizes of VP3-encoding gene and pCI-neo vector, respectively (Supplementary Figure S1). Sequencing results confirmed that VP3-encoding gene was successfully cloned into pCI-neo vector. The predicted 237 amino acids of the VP3 protein showed a molecular mass of about 26 kDa. There are three common potential gylcosylation sites (^32^NLS^34^,^35^NSS^37^, and^200^NSS^202^) (Supplementary Figure S2). Amino acid sequences of VP3 screen reveals that there is a motif rich in basic amino acids (^1^GKRKPCRRPIHKPKN^15^) (eight basic amino acids underlined), which located at the N-terminal region.

### mAbs production and characterization

After cell fusion, the hybridoma cell lines secreting anti-VP3 antibody were screened by ELISA. Three mAbs directed against VP3 were selected for subcloning at least three times. The mAbs were produced and designed as 3B7, 4F8, and 3E9. Hybridomas were selected to produce mAbs in mice, and the ascetic fluids were used for further characterization. The isotypes of the mAbs were IgG1 (3B7) and IgM (4F8 and 3E9). Concentrations of immunoglobulin ranged from 0.71 to 15.83 µg/ml. The neutralization test showed that 3B7 neutralized the DHAV-1 HP1 virus with a neutralization titer of 16. 4F8 and 3E9 did not show any neutralizing activity.

### VP3 protein detection by Western blot

**Figure 1:**
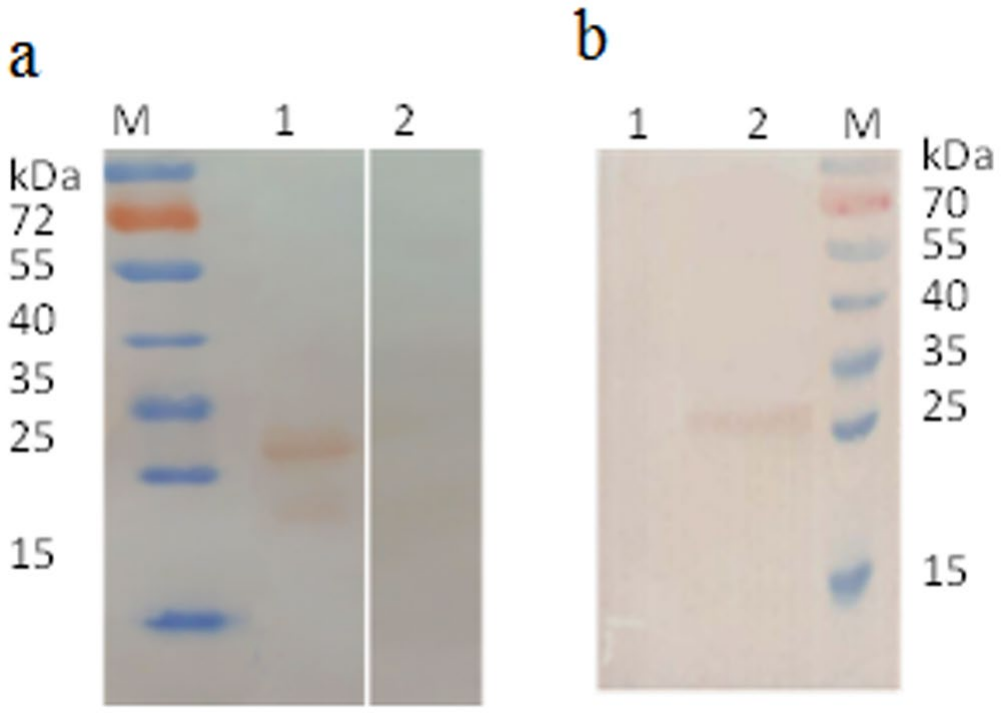
**V**P3 protein detection in pCI-neo-VP3 transfected DEF cells by Western blot. (**a**) VP3 protein detected with duck anti-DHAV-1 serum. Lane M, protein molecular weight marker. 1, representative pCI-neo-VP3 transfected DEF cells. 2, representative pCI-neo transfected DEF cells. (**b**) VP3 protein detected with mAbs. 1, representative pCI-neo transfected DEF cells. 2, representative pCI-neo-VP3 transfected DEF cells. Lane M, protein molecular weight marker.

To prove VP3 expressed in pCI-neo-VP3 transfected DEF cells, duck anti-DHAV-1 sera were used first to detect VP3 protein by Western blot. Representative Fig. 1a showed that duck anti-DHAV-1 sera could react with 26 kDa VP3 protein in pCI-neo-VP3 transfected DEF cells, whereas pCI-neo transfected DEF cells did show any reaction to duck anti-DHAV-1 sera. pCI-neo-VP3 transfected DEF cells were then used to assess whether the obtained mAbs recognize the eukaryotic expressed VP3 protein by Western blot. Representative Fig. 1b demonstrated that three mAbs strong reacted with 26 kDa VP3 proteins in pCI-neo-VP3 transfected DEF cells, whereas pCI-neo transfected DEF cells did not show any reaction.

### Detection of native VP3 protein by immunofluorescence assay

**Figure 2:**
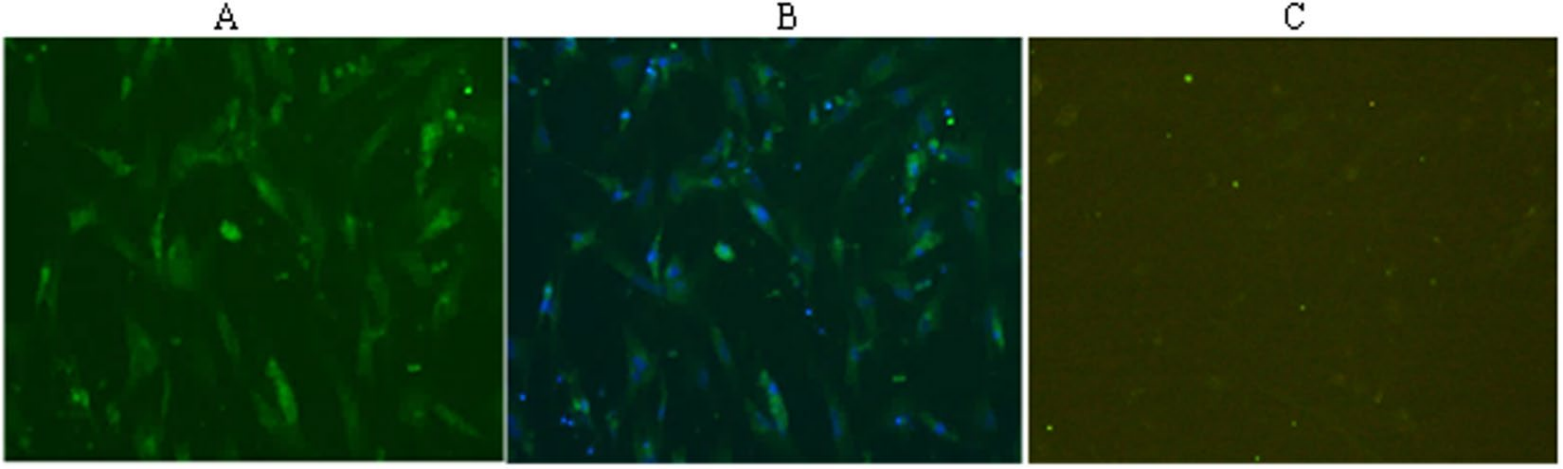
VP3 protein detection in DHAV-1/DHAV-3 infected DEF cells by IFA. (**A**) Representative DHAV-1 infected DEFs detected by mAbs; (**B**) Representative DHAV-1 infected DEF cells detected with mAbs and then stained with DAPI; (**C**) Representative DHAV-3-infected DEF/uninfected DEF cells detected by mAbs (negative control).

An immunofluorescence assay (IFA) was performed on DHAV-1 HP-1/DHAV-3 JT infected DEF to assess whether the produced mAbs recognize the native-form of VP3 protein. Three mAbs strongly reacted with HP-1-infected DEF cells, whereas DHAV-3 JT infected or uninfected DEF cells showed no reaction (represented by Fig. 2). DAPI staining indicated that the VP3 was located in the cytoplasm and nucleus of the DHAV-1 infected DEF cells.

### Epitope Prediction

**Figure 3:**
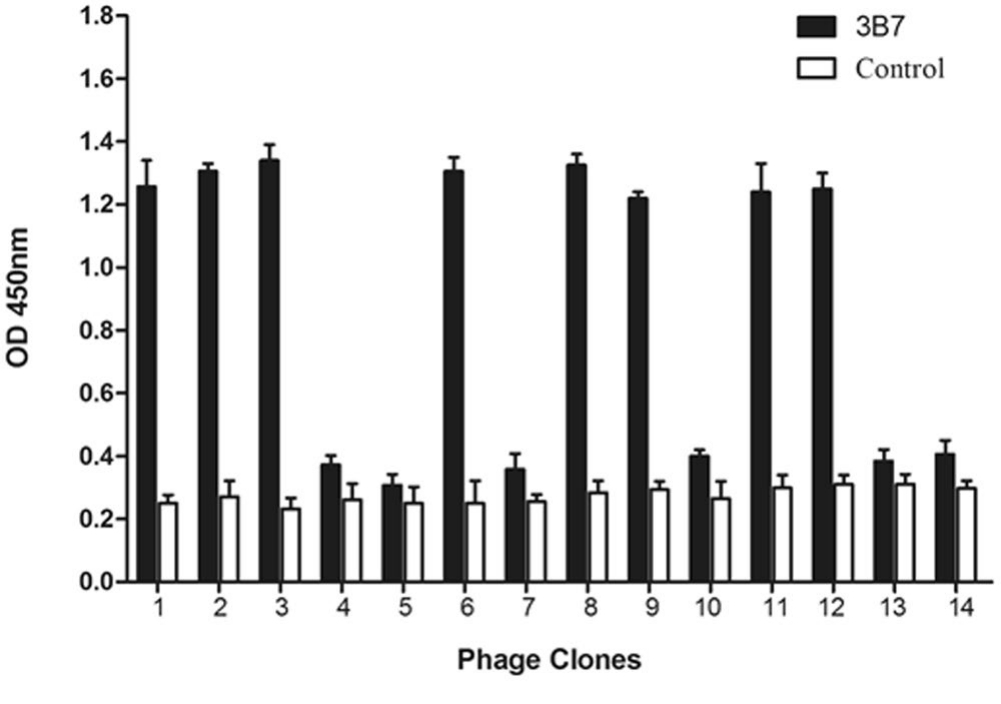
Reactivity of selected phages for mAb 3B7 binding in phage enzyme- linked immunosorbent assay (ELISA). The selected phage clones were detected by mAb 3B7 or the anti‐ porcine interferon (IFN)- c mAb (negative control) after three rounds of iopanning. OD, optical density.

To map the precise epitope location of the VP3, a phage displayed 12-mer random peptide library was screened by using mAb 3B7. After three rounds of biopanning, 14 phage clones were selected and their reactivity to mAb 3B7 was evaluated (anti-porcine IFN-c mAb as the negative control). Eight clones (1–3, 6, 8, 9, 11, and 12) reacted with mAb 3B7 (OD450 nm, >1.20) but did not react with the anti-porcine IFN-c mAb (OD450 nm, <0.3) (Fig. 3). The other 6 clones were less reactive with mAb 3B7 (OD450 nm, OD < 0.37). Eight phage clones with the high OD values were sequenced and consensus sequence PSNM was found (Table 1). Sequence alignment showed that this sequence is quite similar to the VP3 region 205 to 208 (^205^PSNI^208^) of strain HP-1, but ^208^I seems different from the putative binding residue ^208^M for inclusion in the linear epitope. A more reasonable interpretation might be that the Met is selected as an alternative to the Ile at position 208 or perhaps the library itself was biased toward Met. It is necessary to decide whether ^208^I should be included in this epitope.

**Table 1:** Peptide sequences of the selected phage clones.

Phage Clone	Sequence											
1	N	I	G	M	D	Y	T	P	S	N	I	T
2	S	S	I	H	M	N	V	P	S	N	I	Q
3	T	A	P	S	P	H	M	P	S	N	A	T
6	Q	H	M	D	P	Y	V	P	S	N	M	H
8	H	N	A	N	A	Q	P	P	S	N	M	M
9	T	G	H	E	S	T	S	P	S	N	A	I
11	H	P	D	A	R	M	I	P	S	N	M	I
12	T	V	F	A	S	A	P	P	S	N	M	Q
Consensus								P	S	N	M	

### Precise amino acids of the epitope

**Figure 4:**
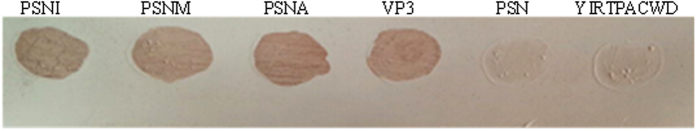
Reactivity of synthesized epitope peptides to mAb 3B7 in dot blotting assay. YIRTPACWD and the VP3 protein were used as the negative and positive control, respectively.

The influence of ^208^I or ^208^A or ^208^M on binding to mAb 3B7 was tested by dot blotting. Dot blotting results showed that the peptide VP3, GST-PSNI, GST-PSNA, and GST-PSNM displayed similar reactivity to mAb 3B7 (Fig. 4), but peptide YIRTPACWD^15^ (as negative control) and GST-PSN did not show any reaction with 3B7, suggesting that ^208^I or ^208^A or ^208^M is necessary and replicable in this epitope and the ^205^PSNI ^208^ should be the minimal B-cell epitope of the VP3 protein of DHAV-1.

### Epitope sequence analysis among DHAV strains

**Figure 5:**
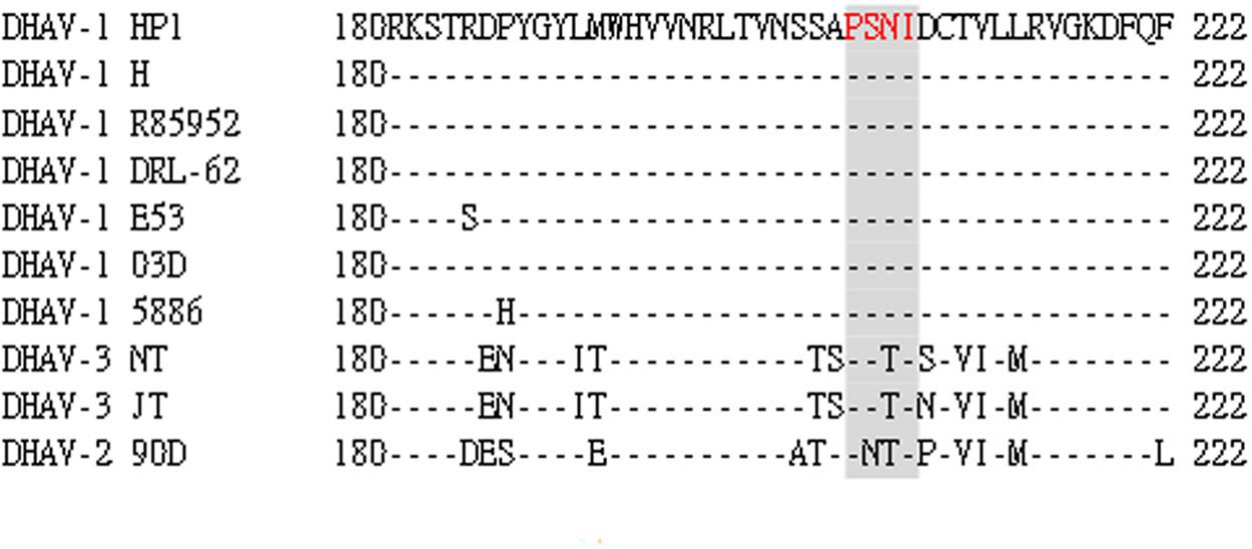
Sequence alignment of the epitope-coding region in the VP3 protein of duck hepatitis type A strains. Amino acid positions for each sequence are numbered. The sequence for the DHAV-1 HP1 strain is shown at the top; the dashes indicate identical amino acids. The identified epitope region is shadowed in red.

To determine whether the PSNI epitope is conserved in the VP3 protein among DHAV, we aligned the VP3 epitope region partial sequence with those of other DHAV-1, DHAV-2, and DHAV-3 sequences available in GenBank (Supplementary Table S1). This sequence alignment revealed that all amino acids in the motif region were identical among DHAV-1 strains (Fig. 5) but quite different from DHAV-2 and DHAV-3, indicating that this motif might be a conserved and specific epitope of the VP3 protein of DHAV-1.

### Immunological reaction of epitope to duck anti-DHAV-1/-DHAV-3 sera

**Figure 6:**
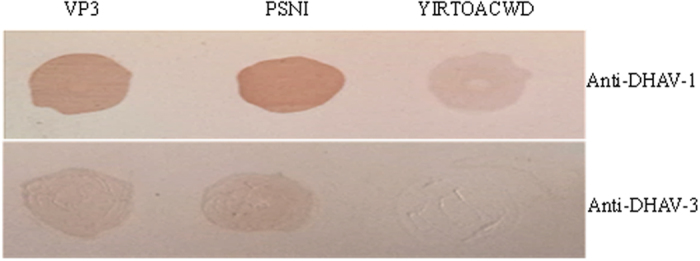
The reactivity of the synthesized epitope peptide to duck anti‐ DHAV-1/-DHAV-3 sera by dot blotting. YIRTPACWD and VP3 protein were used as the negative and positive controls, respectively.

Dot blotting assay was used to test whether the identified motif PSNI recognized by duck anti-DHAV-1/-DHAV-3 sera. Dot blotting showed that the peptide PSNI and VP3 protein of DHAV-1 were recognized by duck anti-DHAV-1 sera (Fig. 6), but did not react with anti-DHAV-3 sera. Negative control peptide (YIRTPACWD) did not show any reaction to duck anti-DHAV-1 or anti-DHAV-3 sera, indicating that the motif PSNI represented a B-cell epitope of the VP3 protein of DHAV-1. Since anti-DHAV-2 serum is not available, the reactivity of DHAV-2 to this epitope cannot be determined.

## Discussion

The use of plasmid DNA as DNA vaccine is a relatively recent approach in the vaccinology field that overcomes many of the problems associated with conventional immunization; for example, the potential danger due to the production and distribution of live vaccine virus. In addition, the proteins expressed by recombinant plasmids are synthesized, processed and presented intracellularly, which is more similar to natural infection than is administration of the conventional vaccine^16^. In this study, to obtain an effective immune response in BALB/c mice, a eukaryotic recombinant plasmid pCI-neo-VP3 as DNA immunogene was successfully constructed, which could synthesize target VP3 protein correctly and efficiently in its native conformation. Three mAbs were prepared and typed using techniques similar to that previously described^17–19^. Western blot showed that the mAbs could specifically react with a 25.5 kDa protein in pCI-neo-VP3 transfected DEF, which was consistent with the expected size of VP3 protein. These results confirmed that pCI-neo-VP3 as DNA immunogen could elicit antibodies against VP3 protein in immunized mice and VP3 protein was successfully expressed in DEF. IFA showed that the VP3 in DHAV-1 infected DEF could also be detected by mAbs, but pCI-neo transfected DEF could not react with mAbs, indicating that mAbs could recognize native VP3 protein of DHAV-1 generated *in vivo*. IFA and DAPI staining showed that the VP3 protein was located both in the cytoplasm and nucleus of DHAV-1-infected DEF cells, which is consistent with the location of VP3 in FMDV infected cells^20^. Most proteins of RNA viruses enter the nucleus via nuclear localization signals (NLSs), which contain a continuous of basic amino acid residues^21–23^; when we scanned the VP3 protein sequence, we noticed multiple basic amino acids (^1^GKRKPCRRPIHKPKN^15^) at the N-terminal of the VP3 protein. Whether this basic motif serves NLS function needs to be proved.

Well-defined epitopes with mAbs provide a platform for studying antigen structure and developing diagnostic reagents and epitope vaccines^24^. Phage-display and peptide screening have been widely used and offer an attractive approach for the identification of epitopes with mAbs. To our knowledge, there have been no previous reports of the linear epitope mapping of the VP3 of DHAV-1. In this study, we identified a conserved epitope between amino acids 205 and 208 on VP3 protein by using phage display system with mAb 3B7. To confirm the essential amino acid residues (amino acid I^208,^ ^205^PSNI^208^) in this epitope region, three designed peptides spanning 205–208 of this epitope coupled with dot blotting analysis demonstrated that motif PSNx is required for mAb 3B7 recognition. Ala or Met selected as an alternative to the Ile at position 208 might be that the library itself was biased toward Met or Ala. This motif is identical to the sequence PSNI of the VP3 protein of DHAV-1. Thus, the peptide PSNI is the minimum motif of the epitope needed to retain maximal binding to mAb 3B7. The peptide was also recognized by DHAV-1 positive duck sera, revealing the importance of the four amino acids of the epitope in antibody-epitope binding reactivity and confirming that PSNI is a DHAV-1 serotype-specific epitope. Further experiments are necessary to explore the functional role of this epitope. Sequence alignments of DHAV-1, DHAV-2, and DHAV-3 strains demonstrated that the motif was highly conserved among DHAV-1, but quite different from DHAV-2 and DHAV-3. This finding supports previous reports that there is no cross reactivity between DHAV-1 and DHAV-2 or DHAV-1 and DHAV-3^2, 4, 5^.

Antibody detection assays based on whole antigens with multiple epitopes show greater sensitivity, but cross-reactions are often observed. Epitopes or mimics of natural antigenic determinants, which mainly originate from dominant responses, favor more highly reactive antigens due to their optimized structure or functional properties. Although the minimal epitope identified in this study has only four amino acids in length, it would be useful in the development of discriminating diagnostic kits for DHAV-1, DHAV-2, and DHAV-3 infection as described previously^25, 26^.

In this study, we prepared mAbs and identified motif PSNI as VP3-specific B-cell epitope with neutralizing mAb 3B7. We could speculate that this neutralization activity resulted from correct folding of the VP3 protein. This finding might be valuable in understanding of the antigenic topology of the VP3 of DHAV-1. The strategy by using eukaryotic recombinant plasmid pCI-neo-VP3 as DNA immunogene to prepare mAbs in mice should be widely used for those proteins with expression and purification difficulties.

## Methods

### Viruses and anti-DHAV-1/-DHAV-3 sera preparation

DHAV-1 HP-1 and DHAV-3 JT strains were propagated in 10-day-old specific-pathogen free (SPF) embryonated or grown in duck embryo fibroblasts cells (DEF) as described previously^5, 10^. Titers of DHAV-1 HP-1 and DHAV-3 JT are 10^-5.5^ and 10^-4.3^ ELD_50_/0.1 mL, respectively. Post infection duck anti-serum against DHAV-1 HP-1 and DHAV-3 JT were prepared as described previously and used for serological assays.

### pCIneo-VP3 construction and transfection in DEF cells

Viral RNA was extracted from DHAV-1 HP-1 by using a viral RNA extraction kit (Qiagen, Shanghai, China). The VP3 encoding gene was amplified by using primers F:5′-TAT CTCGAGCCACCATGGGAAAGAGA AAACCACGC-3′(the *Xho* I site is underlined) and R:5′-AAGCCCGGGTCACTGATTATT GGTTGCCATCTG-3′(the *Sma* I site is underlined) and then cloned into the eukaryotic expression vector pCI-neo (Clontech) to generate pCI-neo-VP3. The correct orientation of the inserts was confirmed by *Xho* I and *Sma* I (New England Biolabs, ON) restriction analysis and nucleotide sequencing. The digested products were analyzed by gel electrophoresis as described previously^26^. Prepared monolayers of DEF cells (approximately 2 × 10^4^ cells) were transfected with 6 µg pCI-neo-VP3 or pCI-neo vector as a negative control as described previously^27^. Transfections were done by using Lipofectamine^TM^ 2000 reagent (Invitrogen) according to the manufacturer’s instructions.

### VP3-specific mAbs preparation and characterization

Plasmid DNA, pCIneo-VP3 was used as an DNA immunogene for development of mAbs in this study. Briefly, five week old female BALB/c mice were subcutaneously primed with 50 µg of plasmid DNA in sterile phosphate-buffered saline (PBS, pH 7.4). The mice received two boosts of 100 µg of plasmid DNA in sterile PBS twice within a 2-week interval. mAbs were produced using techniques similar to that described previously^16–18^. The mice were euthanized by anesthesia and the spleens were removed using aseptic techniques. Splenocytes were fused with Sp2/0 myeloma cells at day 3 after the last boosting. After limiting serial dilutions, hybridoma cells binding to VP3 were selected by enzyme-linked immunosorbent assay (ELISA). Hybridoma cell lines secreting antibodies against VP3 were screened and subcloned at least three times by a limiting dilution method and ascitic fluids were prepared with the cloned hybridoma in BALB/C mice. The study was approved by the Committee of the HVRI of the Chinese Academy of Agricultural Sciences, and all mice were used in accordance with guidelines. Isotypes of the produced mAbs were determined by using Mouse Immunoglobulin isotyping kit (Zymed Laboratories, Inc.) according to the manufacture's instruction. Virus neutralization test was used to test mAbs neutralizing activity to virus. The test was performed using 6-well microplates as described previously^10, 12, 27^. Briefly, 100 μL of serial diluted mAbs were incubated with 100 μL 10^2^ TCID50 of DHAV-1 for 1.5 h at 37 °C. The mixtures were used to inoculate DEF cells. Sera from uninfected healthy mice (diluted in phosphate buffered saline, PBS) and uninfected DEF cells served as controls. Cells were observed daily for cytopathic effects (CPE) for 5 days. Neutralization titers were read as the highest mAb dilution that protected >95% of the cells from CPE.

### Western blot assay

To examine whether anti-VP3 mAbs recognize the VP3 protein in pCI-neo-VP3 (pCI-neo as negative control) transfected DEF, western blot was used to examine the binding ability of duck anti-DHAV-1 serum or mAbs to eukaryotic expressed VP3 proteins. Four days after transfection, collected DEF cells were lysed with RIPA buffer (100 mM Tris-HCl, pH 8.3–8.6, 2% Triton X-100, 150 mM NaCl, 0.6 M KCl, 5 mM EDTA). After centrifugation, the lysate were then subjected to 10% SDS-PAGE and transferred to nitrocellulose membranes. The membranes were probed with duck anti-DHAV-1 serum followed by a secondary HRP-conjugated goat anti-duck antibody (KPL, MD, USA) or mAbs followed by a secondary HRP-conjugated goat anti-mouse antibody (KPL, MD, USA).

### Immunofluorescence assay

The immunofluorescence assays were carried out to test mAb specificity to native form of VP3 protein in DHAV-1 HP-1 or DHAV-1 JT strain as described previously^17^. Briefly, monolayers of DEF (approximately 2 × 10^4^ cells) grown on coverslips in 6 well plates were infected with DHAV-1 HP-1 (10 M.O.I.) as described previously and then incubated at 37 °C for 24 h. After 48 h infection, the infected monolayers or uninfected DEFs (as a negative control) were washed twice with PBS and fixed in methanol at −20 °C for 20 min and then probed with different anti-VP3 mAbs (diluted 40 × ) for 1 h at 37 °C. Blocking was carried out in 10% FBS solution at room temperature for 1 h. After washing for three times, the cells were incubated with fluorescent-conjugated Goat anti-Mouse IgG antibody (KPL, MD, USA) (500 × dilution). To confirm the location of the VP1 protein, the DEF cells were then stained with DAPI as described previously^21^. Bound antibodies and stained cells were visualized under a fluorescence microscope.

### Epitope mapping

Isotypes IgG mAb identified by Mouse Immunoglobulin isotyping kit was used for epitope mapping. mAb 3B7 was first purified from mouse ascites fluid using protein G agarose (Invitrogen), according to manufacturer instructions. The concentration of purified IgG was determined by measuring absorbance at 278 nm. The epitope was mapped with purified mAb 3B7 by using the Ph.D-12TM Phage Display Peptide Library Kit (New England BioLabs), as previously described^12, 28, 29^. Three rounds of biopanning were performed to select phage clones. Briefly, each well of a 96-well plate was coated with 10 μ g/mL of purified 3B7 and incubated with blocking buffer. The phage library was then added to the plate and incubated for 1 hour. After five washes with TBS buffer, 1 M Tris-HCl was added to the plate to elute the bound phages. The phages were then amplified and titred on LB/IPTG/Xgal plates for selection.

### ELISA and sequencing of phage clones

After three rounds of biopanning, as described above and elsewhere^12, 28, 29^, individual phage clones were selected for target binding in an ELISA. Briefly, 96-well plates were coated with 100 ng of mAbs or mouse anti–porcine IFN-c (Sigma-Aldrich) as a negative control. The coated wells were then blocked, and the selected phages were added. The coated plates were then washed ten times with TBST, and bound phages were detected with horse radish peroxidase (HRP)-conjugated sheep anti–M13 antibody (Pharmacia), as described previously^12, 28, 29^. Color development was achieved by adding a substrate solution containing o-phenylenediamine. Positive phage clones were sequenced with M13 primer.

### Sequence analysis

To assess the level of conservation of the epitope among DHAV-1, DHAV-2, and DHAV-3, we performed sequence alignments of the epitope corresponding locations in the VP3 protein of DHAV strains using Lasergene software (DNASTAR)^30^. Representative DHAV-1, DHAV-2, and DHAV-3 virus information used in sequence analyses was listed in Table S1.

### Minimal epitope identification and epitope cross-reactivity to DHAV-1-/DHAV-3-positive serum

To define the fourth amino acid x in PSNx, four GST-taged peptides PSN, PSNI, PSNA, PSNM spanning 205–208 amino acids of VP3 were synthesized (with purity >95%). Briefly, complementary oligonucleotide primers that were specific for each peptide fragments were designed as previously described^28^. Nucleotide segments with sticky ends were produced by *Xho* I/*Sma* I digestion and direct annealing. The oligonucleotide fragments were then cloned into the pGEX6p-1 vector (GE Healthcare). The expressed peptides were purified using a GST Purification Kit (TaKaRa). Dot blotting assay was performed to identify the minimal epitope and epitope peptide cross-reactivity to DHAV-1-/DHAV-3-positive serum. Breifly, approximately 1 μ g of each synthesized peptide or epitope peptide diluted with TNE buffer was spotted onto the nitrocellulose membrane (Millipore) and incubated with 3B7 (diluted 1:1000 in PBS) or with DHAV-1-/DHAV-3 serum (1:200 in PBS) at 37 °C for 1 hour. After washing three times with PBST, the membrane was probed with either HRP-conjugated goat anti–mouse IgG (1:500 dilution, KPL) or HRP-conjugated goat anti–duck IgG (1:500 dilution, KPL) at 37 °C for 1 hour. Peptide YIRTPACWD and VP3 protein were used as negative and positive control, respectively.

## Electronic supplementary material

Supplementary Information 
