# Supplementary material for: Mapping a Type-specific Epitope by Monoclonal Antibody against VP3 Protein of Duck Hepatitis A Type 1 Virus
Source: Sci Rep. 2017 Sep 7;7:10820. doi: 10.1038/s41598-017-10909-7 (PMC5589924; doi:10.1038/s41598-017-10909-7)
Supplement: Supplementary file 1 — Supplementary Information [file 41598_2017_10909_MOESM1_ESM.pdf]

Mapping a Type-specific Epitope by Monoclonal Antibody against VP3 Protein of Duck Hepatitis A Type 1 Virus

Xiaoying Wu[‡], Tingting Zhang[‡], Fanyi Meng[‡], Dongchun Guo, Xiuchen Yin, Shaozhou Wulin, Chenxi Li, Qingshan Zhang, Ming Liu*, Yun Zhang*

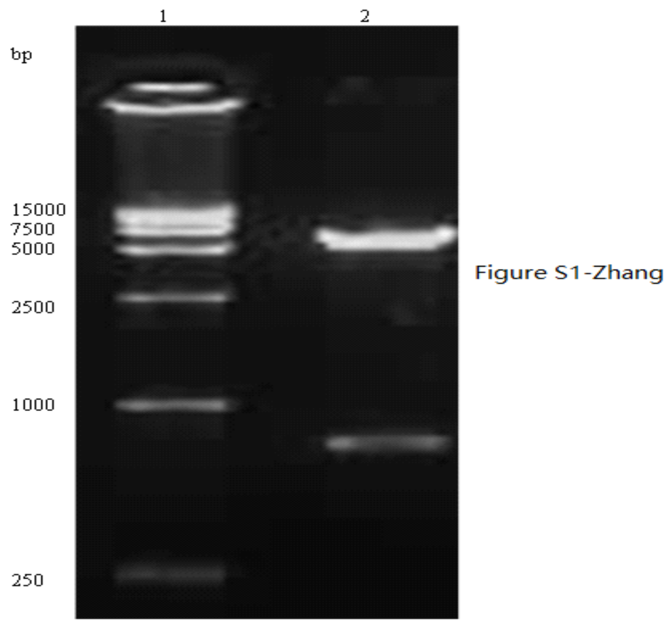

Figure S1. The recombinant pCI-neo-VP3 identification by *Xho* I and *Sma* I digestion. Lane 1, DNA molecular weight marker; 2, pCI-neo-VP3 plasmid digested with *Xho* I/*Sma* I.

```
DHAV-1 HP1     GKRKPRRRPIHKPKNPPQEPRVIIQPGPGAANLSNSSVIAMAESTALANEGTAVDYSTAGCASSVDDVIMVLRRWQILASFQWQSTLTPSARINRYQLIFRNIPTFSLFFDKFQYWRG118
DHAV-1 H       ----------------------------------------------------------------------------------------------------------------------118
DHAV-1 R85952  ----------------------------------------------------------------------------------------------------------------------118
DHAV-1 DRL-62  -----------------------------------------------------------------------------------------V---------S------------------118
DHAV-1 E53     -----------------------------------------------------------------------------------------T----------------------------118
DHAV-1 03D     -----------------------------------------------------------------------------------------V----------------------------118
DHAV-1 5886    ------------I---------------------------------------I------------------------------------V-------V--------------------118
DHAV-3 NT      --K-----------S----S-I----------------VT----V-----------------------V--------VGDS--AN-V--GN--S-F-VV-DRM---A-----------118
DHAV-3 JT      --K-----------S----S-I----------------VT----V--------------R--------V--P-----VGD---AN-V--GN--S-F-VV-NRM---A-----------118
DHAV-2 90D     ------------V-----S-I----------------TL----S-------S-----------I---LV-------IGN-N-AN-S--GD--T---IV-N-M---A-------F----118

DHAV-1 HP1     SLEVKFMTFGSQFNTGRYQMSWYPIADGEQSSAQCQNSVFVTGDVCATPVTLTLPFTSTTWRKSTRDPYGYLMWHVVNRLTVNSSAPSNIDCTVLLRVGKDFQFTAPLYGNLQMATNNQ237
DHAV-1 H       -------------------------------L---------------------------------------------------------------------------------------237
DHAV-1 R85952  -------------------------------L---------------------------------------------------------------------------------------237
DHAV-1 DRL-62  -------------------------V-----L---------------------------------------------------------------------------------------237
DHAV-1 E53     -------------------------------L---------------------------------S-----------------------------------------------------237
DHAV-1 03D     -------------------------------L---------------------------------------------------------------------------------------237
DHAV-1 5886    -------------------------------------------------------------------H---------------------------------------------------237
DHAV-3 NT      -----LL-----------------VSN---TL----------C------A--I----N--------EN---IT-----------TS--T-S-VI-M--------------G----A---237
DHAV-3 JT      -----LL-----------------VSN---TL----------C------A--I----N--------EN---IT-----------TS--T-N-VI-M--------------G----A---237
DHAV-2 90D     -----LL-----------------LGS-NHTL--------------------I----N-------DES----E----------AT--NT-P-VI-M-------L------T----A---237
```

Figure S2. Sequence alignment of the VP3 protein of duck hepatitis type A strains. Amino acid positions for each sequence are numbered at right. The sequence for the DHAV-1 HP1 strain is shown at the top; the dashes indicate identical amino acids. The putative NLS region is shadowed. Potential gylcosylation sites in red.

Table S1. **Virus strains information used in sequence analyses**

Species (Sero type)	Strain	GenBank no.	Isolation
Duck hepatitis A virus (DHAV-1)	HP	EF151312	China
Duck hepatitis A virus (DHAV-1)	E53	EF151313	China
Duck hepatitis A virus (DHAV-1)	DRL-62	DQ219396	America
Duck hepatitis A virus (DHAV-1)	R85952	DQ226541	America
Duck hepatitis A virus (DHAV-1)	H	DQ249300	UK
Duck hepatitis A virus (DHAV-1)	5886	DQ249301	America
Duck hepatitis A virus (DHAV-1)	03D	DQ249299	Taiwan
Duck hepatitis A virus (DHAV-2)	90D	EF067924	Taiwan
Duck hepatitis A virus (DHAV-3)	NT	KU860090.1	Viet Nam
Duck hepatitis A virus (DHAV-3)	JT	JF835025	China
